# Case report: First case of early adenoid cystic carcinoma and squamous cell carcinoma collision cancer treated by endoscopic submucosal dissection

**DOI:** 10.3389/fonc.2023.1072336

**Published:** 2023-02-02

**Authors:** Zheng Liang, Yongqiu Wei, Peng Li, Rui Xu, Qiaozhi Zhou, Shutian Zhang

**Affiliations:** ^1^ Department of Gastroenterology, Beijing Friendship Hospital, Capital Medical University, National Clinical Research Center for Digestive Disease, Beijing, China; ^2^ Department of Pathology, Beijing Friendship Hospital, Capital Medical University, Beijing, China

**Keywords:** collision cancer, early esophageal cancer, esophageal squamous cell carcinoma, esophageal adenoid cystic carcinoma, endoscopic submucosal dissection

## Abstract

**Background:**

Collision cancer, a rare tumor, rarely occurs in the esophagus. Most reported cases of esophageal collision cancers are advanced cancers that can only be treated with surgery or palliative chemoradiotherapy. Here, we report a rare case of collisional squamous cell carcinoma (SqCC) and adenoid cystic carcinoma (AdCC) that was detected in the early stages by endoscopy.

**Case summary:**

A 66-year-old man presented with retrosternal pain after swallowing and underwent endoscopy. Pathological biopsy showed high-grade squamous intraepithelial neoplasia. The lesion was removed by endoscopic submucosal dissection (ESD) after magnification and endoscopic ultrasonography. Postoperative pathology proved that the lesion was collision cancer comprising SqCC and AdCC. After six months of postoperative follow-up, there was no recurrence of esophageal cancer.

**Conclusions:**

We provided a case report related to the diagnosis and treatment of esophageal collision cancer, especially early collision cancer. More research is needed to provide insights into the management of collision cancers.

## Introduction

Collision cancer refers to a tumor that occurs at the same site but originates from two tissues that infiltrate each other but do not migrate to each other (1). Collision cancer has been reported in many sites of the human body, including the skin, crania, lung, bladder and uterus (2–4). In the digestive system, collision cancer mostly occurs in large digestive glands, such as the liver and pancreas (5, 6). Approximately 2.0% to 3.6% of collision cancers occur in the liver, and approximately 0.06% to 0.2% occur in the pancreas (7, 8). In contrast, this type of tumor is rarer in the digestive tract. A previous review reported 53 cases of collision cancers of the esophagus, stomach, small intestine and large intestine (9).

To date, 16 cases of esophageal collision cancer have been reported in the English literature (10–19). However, most previous reports on esophageal collision cancer described advanced cancer with tumor tissue invading the muscle layer, and all previously reported cases of esophageal collision cancer have been treated with radical surgical resection or palliative chemoradiotherapy. The collision of squamous cell carcinoma (SqCC) and small cell carcinoma (SmCC) was the most common combination.

Here, we report a rare case of a 66-year-old Chinese man with collisional cancer of SqCC and adenoid cystic carcinoma (AdCC), which was detected in the early stages by endoscopy. This is the first case of early esophageal collision cancer that was removed by endoscopic submucosal dissection (ESD) and had not recurred at 6 months of follow-up.

## Case description

The patient was a 66-year-old man who was admitted to Beijing Friendship Hospital mainly because of retrosternal pain during swallowing for more than six months. The main symptom was a stabbing pain in the chest behind the breastbone when swallowing solid food, which was relieved after swallowing. He did not report dysphagia, acid regurgitation, heartburn, nausea, vomiting, or melena. There was no significant change in body weight in the past six months. In terms of past history, the patient had a smoking history of more than 30 years, approximately 30 cigarettes per day, and had quit smoking for 4 years. The patient had no other underlying diseases and no family history of cancer. Physical examination revealed no obvious abnormality.

The patient underwent electronic endoscopy in a local hospital on February 22, 2022. Flaky erosions were observed 29 cm away from the incisors, and they were approximately 0.8*1 cm in size with surfaces covered with white hair. A biopsy was taken from the erosion site, and the local hospital’s pathology suggested high-grade squamous intraepithelial neoplasia.

After admission, the patient’s routine blood, liver and kidney function, electrolytes, myocardial enzymes and other laboratory tests showed no abnormalities. Only the tumor marker prostate-specific antigen was increased. Enhanced chest computed tomography showed no esophageal space-occupying lesions or swollen lymph nodes around the esophagus.

**Figure 1:**
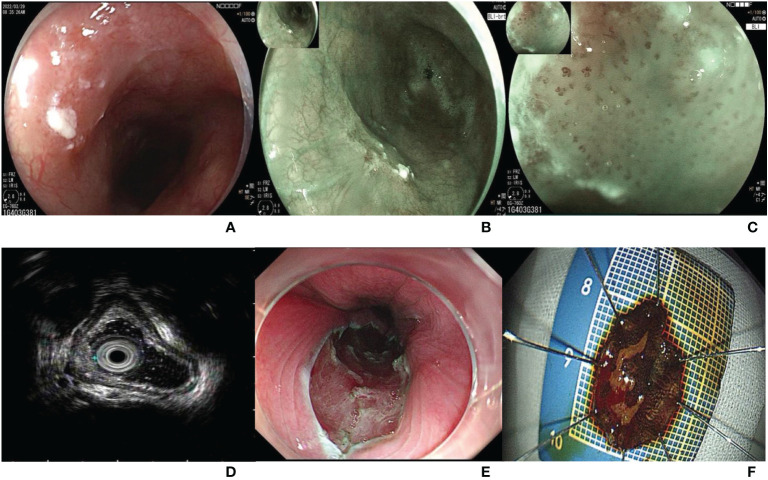
**(A)** A type 0-IIa lesion under white light endoscopy, approximately 1*1 cm in size, located in the middle of the esophagus and 28-29 cm from the incisors; **(B)** Esophageal lesion under blue laser endoscopy; **(C)** The background staining was positive under blue laser imaging magnifying endoscope. The JES type was B1, and the AVA type was small AVA. **(D)** Five-layer structure of the esophageal wall at the lesion was clear, and the mucosal layer was slightly thickened under ultrasound endoscope. **(E)** Esophageal wound after ESD. **(F)** An 18*14 mm esophageal mucosal tissue.

The patient underwent endoscopy in our hospital on March 29, 2022. A type 0-IIa lesion, approximately 1*1 cm in size, was located in the middle of the esophagus and 28-29 cm from the incisors (**Figure 1A**). The lesion mucosa was red and rough, with good extension. The lesion boundary was clear under white light observation, and the lesion mucosa did not stain with 1.25% iodine staining. Blue laser imaging magnifying endoscopy (BLI-ME) revealed positive background staining (**Figures 1B, C**). The Japan esophageal society (JES) type was B1, and the avascular area (AVA) type was small AVA. Endoscopic ultrasonography suggested that the five-layer structure of the esophageal wall at the lesion was clear, and the mucosal layer was slightly thickened (**Figure 1D**). ESD was performed to remove the diseased mucosa, and 18*14-mm esophageal mucosal tissue was obtained (**Figures 1E, F**). There were no short-term complications, such as bleeding, perforation or infection, after endoscopic surgery.

**Figure 2:**
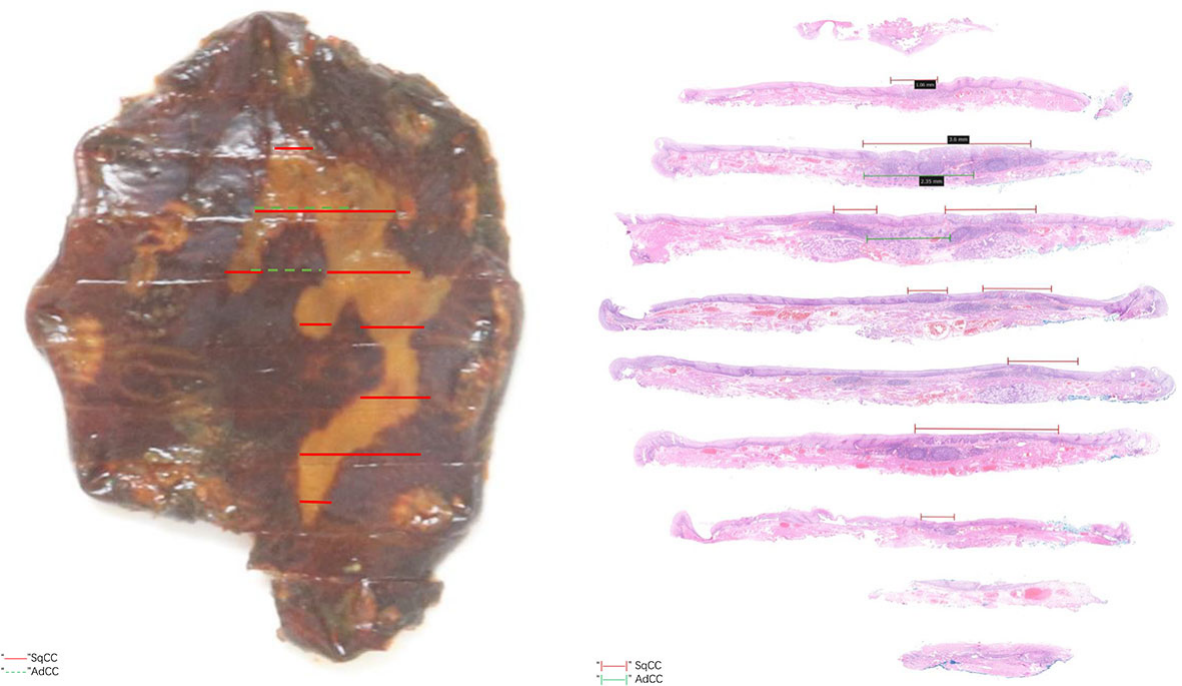
Translucent observation of the lesion and microscopic appearance of the lesion.

**Figure 3:**
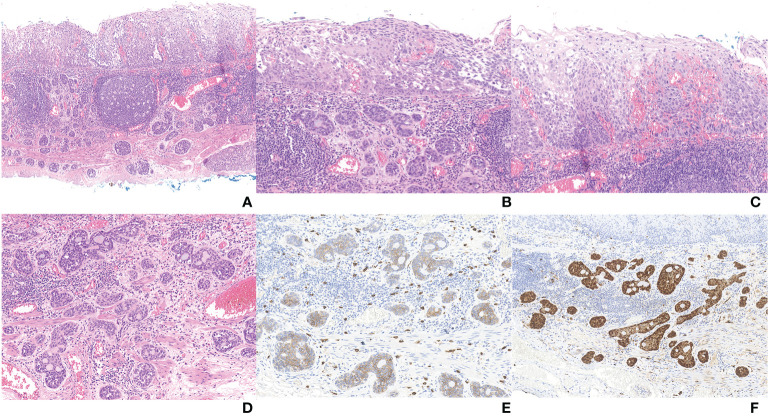
**(A)** Full view of collision carcinoma; **(B)** Area of collision between SqCC (up) and AdCC (down); **(C)** Area of SqCC; **(D)** Area of AdCC; **(E)** Immunohistochemical staining of AdCC (CD117); **(F)** Immunohistochemical staining of AdCC (S-100).

The postoperative pathological results showed that there were two malignant tumor components in the tissue submitted for examination (**Figures 2**, **3A**). And microscopy revealed an abrupt transition between these two components that developed adjacently but never intermingled (**Figure 3B**). Combined with preoperative evaluation that failed to confirm any primary lesions that had metastasized to the esophagus, this patient was unequivocally diagnosed with an esophageal collision tumor. One of them was SqCC (approximately 4*2 cm in area) (**Figure 3C**). The cancer tissue infiltrated the lamina propria (pT1a-LPM). Another cancerous tissue was tubular and had cribriform structures with variably solid components (**Figure 3D**), which was located in the lamina propria and submucosa, with an infiltration depth of 60 μm into the submucosa (pT1b-SM1). Subsequent immunohistochemistry of this component yielded positive CD 117, S-100, p63, and CK8 staining (**Figures 3E, F**). Using the World Health Organization’s classification of tumors of the digestive system 2019, we diagnosed this component as an AdCC (14*4 mm in area) (20). The horizontal resection margin was clean, and the tumor was approximately 20 microns away from the nearest vertical resection margin. This lesion was free of lymphovascular invasion.

Since the lesion had clean incisors and no vascular infiltration, additional surgery or chemoradiotherapy was not considered. After 6 months of follow-up, the patient did not complain of any discomfort. Three months after surgery, endoscopy revealed that there was a white scar approximately 28 to 29 cm away from the incisors in the middle esophagus, and esophageal stenosis and esophageal fistula were not observed. No abnormality was observed by narrowband light imaging (NBI), and no light staining was found after staining with 1.25% iodine solution.

## Discussion

A collision tumor is a subtype of neoplasm consisting of two or more distinct cell populations, and some other types include composite tumors (no clear-cut interface or a transition zone between histological patterns) and carcinosarcomas (extensive intermingling between cell populations) (10). Furthermore, amphicrine neoplasms, one cell population exhibiting characteristics of both epithelial and sarcomatous cells, and cancer-to-cancer metastasis are also included in these rare neoplasms (21, 22). The diagnostic criteria of collision tumors proposed in the previous literature are as follows: a. two distinct topographically separate sites of origin for the two components must be present; b. there must be at least some separation of the two components so that, despite intimate mixing at points of juxtaposition, a dual origin can still be recognized; and c. at the areas of collision, in addition to intimate mixing of the two components, some transitional patterns may be seen (23). Combined with the results of immunohistochemistry, the pathological diagnosis of what we reported was that of an AdCC associated with an SqCC with features of a collision tumor.

According to the definition of collision cancer, the previous literature was strictly searched. As of August 31, 2022, a total of 16 cases (10–19) of esophageal collision cancer have been reported in the English literature (**Table 1**). Notably, most of the literature reports were from East Asia, and only 2 cases were from European and American countries. This may be related to the lower incidence of esophageal cancer in Western countries and the predominant pathological type of esophageal adenocarcinoma (24), while most esophageal collision tumors contain elements of squamous cell carcinoma. The incidence of esophageal collision cancer was higher in males than in females, with a male-to-female ratio of 13:3, mostly in the 60-70 years old age group. This distribution was similar to that of normal esophageal cancer.

**Table 1:** Clinical Characteristics, Pathology, and Treatment Options of Esophageal Collision Tumors.

Author, year	Country	Age	Sex	Location	Gross type	Pathology of biopsy	Pathology of surgery	Pathologic stage	Surgical therapy	Overall survival
Schizas,2017	Greece	76	Male	Middle 1/3	Protuberant	ADC	SmCC+ADC+SRCC	pT3N1M0	+	6 months/alive
Yao,2015	China	55	Male	Lower 1/3	NA	NA	SqCC+LMS	pT3N0M0	+	60 months/alive
Choe,2020T	Korea	64	Male	Lower 1/3	Ulcerative	SqCC+SmCC	SqCC+SmCC	pT3N3M1	–	24 months/died
Li,2013	China	66	Male	Lower 1/3	Ulcerative	SmCC	SqCC+SmCC	pT1bN1M0	+	18 months/alive
Adachi,2014	Japan	62	Male	Middle 1/3	Medullary	ASC	SqCC+SmCC	pT1bN0M0	+	NA
Qian,2014	China	69	Male	Middle 1/3	Ulcerative	NA	SqCC+GIST	pT3N3M0	+	NA
Wilson,2000	America	51	Male	Lower 1/3	Protuberant	ADC	ADC+SmCC	NA	+	NA
Wang,2014	China	60	Male	Middle 1/3	Ulcerative	SqCC	SqCC+SmCC	pT3N2M0	+	17 months/died
Wang,2014	China	66	Male	Lower 1/3	Protuberant	SmCC	SqCC+SmCC	pT1bN1M0	+	12 months/alive
Wang,2014	China	57	Female	Middle 1/3	Protuberant	SqCC	SqCC+AdCC	pT1bN0M0	+	8 months/alive
Kang,2020	Korea	70	Male	Middle 1/3	Ulcerative	SqCC	SqCC+SmCC	pT1bN0M0	+	NA
Zhang,2020	China	72	Male	Lower 1/3	Protuberant	ADC	SqCC+ADC	pT4N1M0	+	17 months/died
Zhang,2020	China	75	Male	Lower 1/3	Medullary	SqCC	SqCC+ADC	pT4N1M1	+	49 months/died
Zhang,2020	China	62	Male	Middle 1/3	Medullary	SqCC	SqCC+SmCC	pT4N2M0	+	13 months/died
Zhang,2020	China	64	Female	Middle 1/3	Ulcerative	SmCC	SqCC+SmCC	pT2N0M0	+	78 months/died
Zhang,2020	China	57	Female	Middle 1/3	Ulcerative	SqCC	SqCC+SmCC	pT1N0M0	+	48 months/alive

SmCC, small cell carcinoma; SqCC, squamous cell carcinoma; ADC, adenocarcinoma; AdCC, adenoid cystic carcinoma; GIST, gastrointestinal stromal tumor; ASC, adenosquamous carcinoma; NA, not available. "+", Undergone a surgical operation, "-", Did not undergo surgery.

The correct diagnosis of a collision tumor is difficult but crucial because individualized treatment and disease monitoring depend on the diagnosis. The medical history, clinical manifestations, and imaging findings of the collision tumor were not specific. The gold standard for routine tumor diagnosis (endoscopic pathological biopsy) yields the diagnosis of only one cancerous component in most cases; accordingly, our literature review yielded only 1 case (12) wherein a collision tumor was confirmed by endoscopic biopsy of the two components. Hence, it is important to examine multiple tumor biopsy sites to improve the efficacy of preoperative diagnosis. However, the difficulty of subsequent treatment due to fibrosis of the esophageal mucosa caused by multiple biopsies must be considered. Immunohistochemistry is a routine method for pathological diagnosis. If immunohistochemistry remains inconclusive, molecular genetic analysis may be an important supplementary method for the diagnosis of collision tumors (25). Fukui et al. used gene sequencing to identify collision tumors and compound tumors (26).

To date, surgery remains the first-line treatment for patients with esophageal collision tumors, as is common for esophageal cancer. However, the presence of multiple components of collision tumors significantly alters treatment options, as it affects the adjuvant treatment options (10). No established guidelines are available. Some papers have argued that treatment should target the more aggressive component, while others consider that combined therapy targeting both tumor components can also be considered (27–29). More evidence is needed to determine the best individualized treatment for collisional tumors. The preoperative pathology of the case reported herein suggested high-grade intraepithelial neoplasia, which led us to use ESD. Postoperative pathological specimens were incidentally obtained as collision cancer. This contingency resulted in the current case being the only case of esophageal collision carcinoma that was removed endoscopically.

Notably, in this case, adenoid cystic carcinoma was one of the two cancerous components. This cancer is very rare in the esophagus, accounting for approximately 0.04%-0.16% of esophageal malignant tumors (30, 31). Due to the morphological similarity, the nomenclature of salivary tumors is adopted (32). AdCC is a type of submucosal tumor (SMT). The diagnosis of SMTs by endoscopic ultrasonography and the choice of ESD to remove submucosal lesions are controversial. He et al. (33) reported 224 upper gastrointestinal SMT patients detected with endoscopy who were further checked by EUS before receiving a series of ESD treatments; these patients also completed 3- and 12-month follow-up EUS detection. The accuracy rate of EUS in pathological diagnosis or the original layer was 82.6% (185/224) or 74.6% (167/224), respectively, and the ESD success rate was 92.9%. Residual tumors were detected with EUS in 3 patients (1.3%) at the 3-month follow-up, and no recurrence was observed during the 12-month follow-up period. Hence, endoscopic ultrasonography appears to be an effective routine follow-up for SMTs in the future, although the health and economic impacts of this measure remain unclear.

In summary, we described the clinical, histologic, and molecular features of a rare collision tumor comprising SqCC and AdCC, which was the first early esophageal collision tumor to be resected endoscopically. There was no recurrence after 6 months of follow-up. We provided more evidence for the diagnosis and treatment of esophageal collision cancer, especially early collision cancer.

## Data availability statement

The original contributions presented in the study are included in the article/supplementary material. Further inquiries can be directed to the corresponding authors.

## Author contributions

QZ, PL, and SZ treated the patient. ZL, YW, and RX wrote the paper. All authors contributed to the article and approved the submitted version.
